# Quality of reporting of clinical non-inferiority and equivalence randomised trials - update and extension

**DOI:** 10.1186/1745-6215-13-214

**Published:** 2012-11-16

**Authors:** Petra Schiller, Nicole Burchardi, Michael Niestroj, Meinhard Kieser

**Affiliations:** 1 Institute of Medical Biometry and Informatics (IMBI), University of Heidelberg, Im Neuenheimer Feld 305, Heidelberg, D-69120, Germany; 2 Coordinating Center for Clinical Trials (KKS), Marburg, Germany

**Keywords:** Reporting quality, Methodological quality, Non-inferiority, Equivalence, Randomised clinical trials

## Abstract

**Background:**

Non-inferiority and equivalence trials require tailored methodology and therefore adequate conduct and reporting is an ambitious task. The aim of our review was to assess whether the criteria recommended by the CONSORT extension were followed.

**Methods:**

We searched the Medline database and the Cochrane Central Register for reports of randomised non-inferiority and equivalence trials published in English language. We excluded reports on bioequivalence studies, reports targeting on other than the main results of a trial, and articles of which the full-text version was not available. In total, we identified 209 reports (167 non-inferiority, 42 equivalence trials) and assessed the reporting and methodological quality using abstracted items of the CONSORT extension.

**Results:**

Half of the articles did not report on the method of randomisation and only a third of the trials were reported to use blinding. The non-inferiority or equivalence margin was defined in most reports (94%), but was justified only for a quarter of the trials. Sample size calculation was reported for a proportion of 90%, but the margin was taken into account in only 78% of the trials reported. Both intention-to-treat and per-protocol analysis were presented in less than half of the reports. When reporting the results, a confidence interval was given for 85% trials. A proportion of 21% of the reports presented a conclusion that was wrong or incomprehensible. Overall, we found a substantial lack of quality in reporting and conduct. The need to improve also applied to aspects generally recommended for randomised trials. The quality was partly better in high-impact journals as compared to others.

**Conclusions:**

There are still important deficiencies in the reporting on the methodological approach as well as on results and interpretation even in high-impact journals. It seems to take more than guidelines to improve conduct and reporting of non-inferiority and equivalence trials.

## Background

With an increasing number of available effective interventions, the conduct of placebo-controlled clinical trials is for many diseases no longer ethically justifiable. New treatments can often not be expected to enhance the efficacy of a standard therapy. However, there are frequent instances in which one is nevertheless interested in evaluating a new therapy because of an expected advantage other than with respect to efficacy. Non-inferiority trials are performed in such situations to rule out that the treatment under investigation has unacceptably worse efficacy than an active control standard therapy, while superiority is acceptable or even desired. In contrast, it is the aim of equivalence trials to demonstrate that the difference between the two treatments is not large in either direction. Non-inferiority and equivalence trials require tailored methodology and raise special challenges with respect to design, conduct, analysis and interpretation [1-5] As a consequence, reporting these trials is also an ambitious task.

The CONSORT statement, which was first published in 1996 and updated in 2001 [6] and 2010 [7], contains a checklist with a set of recommendations for reporting randomised controlled clinical trials to support authors and editors. Recent publications indicate that the reporting quality has somewhat improved over time and that the CONSORT statement accounts for a substantial part in this process [8,9]. Nevertheless, evaluation of the reporting quality of specific trial designs indicated substantial deficits in the reporting of conduct, analysis and results [10,11]. In a comprehensive review, Le Henanff *et al*. assessed the methodological quality of 162 articles on non-inferiority and equivalence trials published in 2003 and 2004 [10]. They found important shortcomings in reporting of such trials and gave practical recommendations for an improvement. At the same time, an extension of the CONSORT Statement was published that focused on the methodological aspects specific to non-inferiority and equivalence trials with the aim of improving reporting [12]. In our study, we describe how non-inferiority and equivalence trials published after the release of the CONSORT extension were conducted and reported, and the extent of adherence to the CONSORT criteria. To this end, we systematically searched and reviewed articles on trials that aimed at demonstrating non-inferiority or equivalence published in 2009. In an additional analysis, we compared the results with those previously established for trials published in 2003 and 2004 [10]. Bioequivalence studies were not included in our investigation as they show several special features making them different from clinical non-inferiority and equivalence trials, such as, for example, inclusion of healthy volunteers, application of equivalence margins that are broadly accepted by regulating agencies and the scientific community, disproportionately frequent use of cross-over design, and conduct under highly standardised conditions. Therefore, a number of topics assessed in our review do not apply, or are not directly comparable with the situation in bioequivalence trials. Furthermore, other similar studies [10,13,14] also excluded bioequivalence trials, and it was one of the aims of our work to compare our results with others.

## Methods

### Search strategy

**Figure 1:**
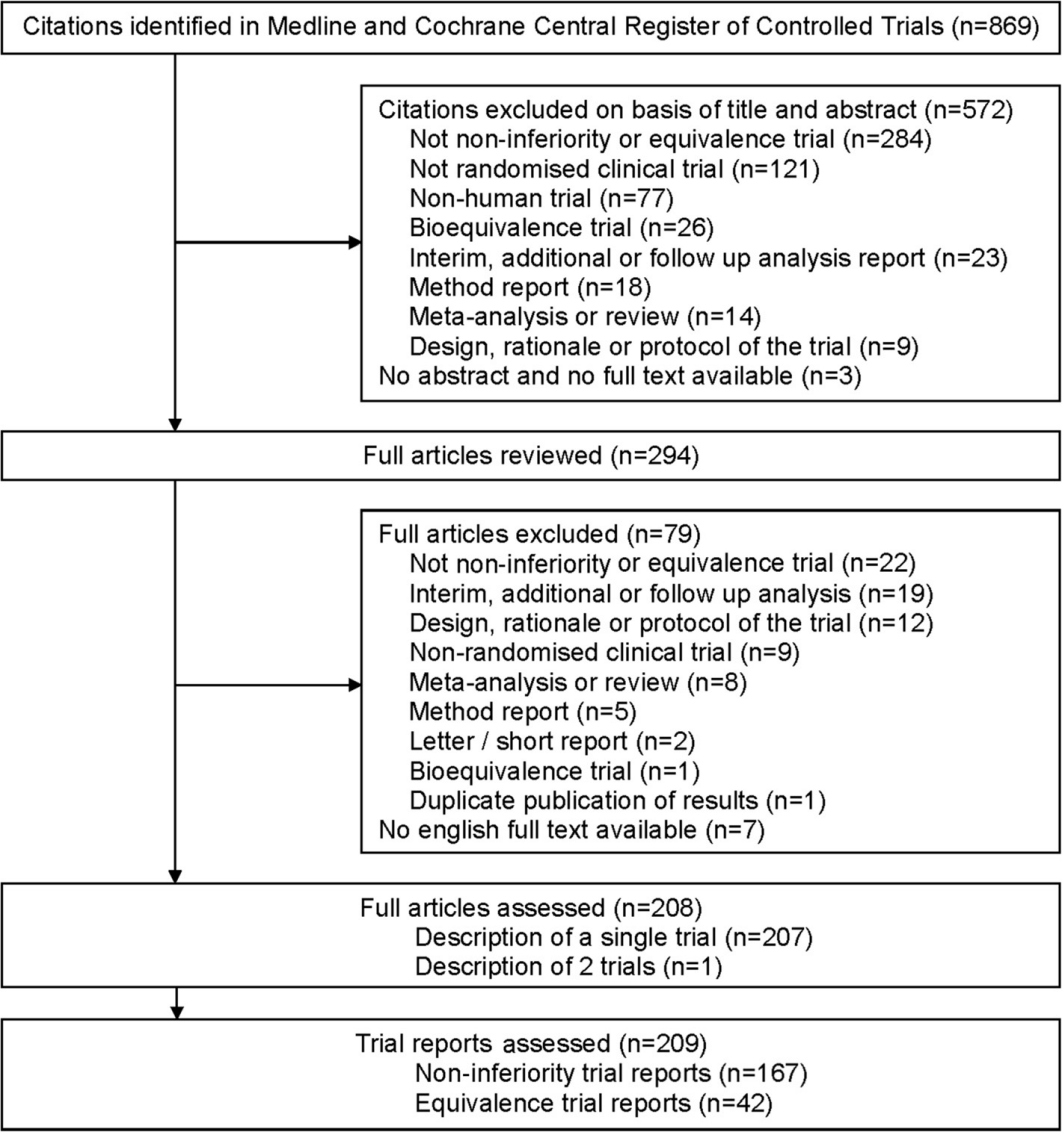
**Identification of reports on non**-**inferiority and equivalence trials.**

We used a computerised literature search of the Medline databases and Cochrane Central Register of Controlled Clinical Trials via Ovid SP to identify reports of randomised non-inferiority and equivalence trials. We defined the following search terms: random* AND (equivalence OR equivalent OR noninferiority OR noninferior OR non-inferiority OR non-inferior) and excluded the publication type Meta-Analysis, Review, and Research Support. The search was limited to citations published between 1 January 2009 and 31 December 2009 in the English language. The due date for the search was 6 April 2010. We selected citations by screening title and abstract to identify relevant reports. The final decision was made on the basis of the full text. After internal harmonisation based on the assessment of the first 25 abstracts, each of the three reviewers (PS, MN, and NB) screened one third of the abstracts to identify the relevant reports. Reasons for exclusion of citations or articles, respectively, are given in Figure 1. In case of duplicate publications only the article that reported the main results for the primary endpoint was selected.

### Evaluation criteria and data extraction

We extracted specific criteria to examine whether reports were prepared in compliance with the extension of the CONSORT statement for the reporting of non-inferiority and equivalence randomised trials [12]. This included criteria referring to the reporting and to the methodological quality of the trials. In addition, we extracted general aspects to characterise the reported trials.

The reference to the respective item described in the CONSORT extension list and the way we abstracted them are given in Table 1. Some of the 22 items listed in the CONSORT extension were difficult to standardise for extraction, or the assessment would have required the evaluation of a number of associated trials published elsewhere. Therefore, we decided to assess 15 derived items that were essential in the context of non-inferiority and equivalence, as well as feasible with regard to the extraction procedure. In addition, we assessed the interpretation given in the reports in relation to the results presented. We also checked whether the relevant guidelines such as the CONSORT extension for reporting of non-inferiority and equivalence randomised trials [12], the revised CONSORT statement [6], and the Points to Consider on the Choice of Noninferiority Margin [15] were referenced.

**Table 1:** **Description of the assessment of CONSORT criteria recommended in the extension to CONSORT for non**-**inferiority and equivalence trials **[11] (**R = reporting item, M = methodological item, I = interpretation**)

**CONSORT criteria**	**Included in present evaluation**	**Category**	**Description of assessed variables**
Specify that the trial is a non-inferiority or equivalence trial	Yes	R	Clearly identified as non-inferiority or equivalence trial in title, abstract or full paper
Rationale for using a non-inferiority or equivalence design	Yes	R	Justification stated for using a non-inferiority or equivalence design
Eligibility for participants with respect to trials that established efficacy of the reference treatment	No	-	-
Interventions intended for each group with respect to trials that established efficacy of the reference treatment	No	-	-
Specific objectives and hypothesis concerning non-inferiority or equivalence	Yes	R	Hypothesis stated clearly (text or formula)
		M	Margin defined
Clearly defined primary and secondary outcome measures with respect to trials that established efficacy of the reference treatment	(Yes)	R	Primary outcome identified clearly (not evaluated whether outcome is identical to those in any trial that established efficacy of the reference treatment)
Sample size calculation using a non-inferiority or equivalence criterion and specifying the margin with the rationale for its choice. When applicable, explanation of any interim analyses and stopping rules (and whether related to a non-inferiority or equivalence hypothesis)	Yes	R	Sample size calculation presented
		R	Elements for recalculation of sample size reported
		M	Margin considered
		R	Justification for margin stated
		R	Interim analyses planned
Method used to generate random allocation sequence including details of any restriction	Yes	R	Method of randomisation reported
		R	Restriction method reported (blocking/stratification/minimisation)
Method used to implement allocation concealment	No	-	-
Who generated the allocation sequence and enrolled and assigned participants	No	-	-
Whether participants, those administering the interventions and those assessing the outcome were blinded to group allocation	Yes	R	Method of blinding reported (any blinding; single blind, double blind, open or double dummy design)
Statistical methods used to compare groups for primary outcome specifying whether a 1- or 2-sided confidence interval approach was used. Methods for additional analyses (subgroups, adjusted analyses)	Yes	R	Statistical methods used for comparison reported
Participant flow through each state of the trial (diagram strongly recommended)	Yes	R	Diagram of flow of participants presented
Dates defining the periods of recruitment and follow-up	Yes	R	Dates reported
Baseline information for each group	Yes	R	Baseline information presented for each group
Number of participants in each group included for each analysis and whether intention-to-treat (ITT) and/or alternative analyses were conducted	Yes	R	Number of participants reported - similar to 13
		R	Analysis sets reported
		M	Results of ITT and per-protocol analysis presented
For each outcome, a summary of results for each group and the estimated effect size and its precision (useful: figure showing confidence intervals and margins)	Yes	M	Results presented using a confidence interval
		R	Report of confidence level and 1- or 2-sided
		R	Report of *P*-value
		R	Figure presented
Address multiplicity by reporting any other analyses performed	No	-	-
All important adverse events or side effects in each group	Yes	R	Adverse events reported
Interpretation of the results taking into account the non-inferiority or equivalence hypothesis, sources of potential bias or imprecision	Yes	I	Interpretation of results presented
			Interpretation correct (non-inferiority/equivalence/superiority/inferiority/inconclusive result/wrong/incomprehensible by means of presented results)
			Statement on expected advantage
Generalisability (external validity) of the trial findings	No	-	-
General interpretation of the results in the context of current evidence	No	-	-

In addition, we compared our results for trials published in 2009 with the results found for non-inferiority and equivalence trials published in 2003 and 2004, before the CONSORT extension was released [10]. In this comparison, we looked for changes in trial characteristics as well as in the adherence to criteria related to reporting and methodological quality. For a further differentiated assessment, we compared the quality of reporting of trials published in four high-impact general medical journals (JAMA, NEJM, Lancet and BMJ; selection such as in Ghimire 2012 [16]) with that of trials published in low-impact general medical journals and in speciality journals.

The data extraction was done by the three reviewers. We developed a form to extract details of the selected articles. At first, the reviewers completed data extraction for a random sample of seven articles. All discrepancies were discussed in two meetings, the data extraction form was modified and the procedure was repeated. The finally agreed procedure was fixed in an instruction form accompanying the data extraction form to improve interobserver agreement. Interobserver agreement was tested using a random sample of 21 articles. Each reviewer independently extracted data from 14 articles. Each subset of seven articles was extracted in parallel by one of the other two reviewers.

The data of all remaining articles were then extracted by a single reviewer. In case of uncertainties with a specific report, a second reviewer checked the data extraction, and a solution was found by discussion.

We classified a trial as non-inferiority or equivalence trial if the terms non-inferiority or equivalence were part of the title or were mentioned as the aim of the trial in the abstract. If this information was missing, the aim of the planned analysis reported by the authors, or the kind of analysis which was actually done, was taken as an indicator for the classification of the trial.

### Data analysis

We calculated descriptive summary statistics for the general and specific items stratified by the trial design. Categorical variables were described by absolute and relative frequency. Continuous variables were described by the median and 25% and 75% percentiles. Differences in trial characteristics and in adherence to reporting criteria (before and after CONSORT extension, high- and low-impact general medical journals and speciality medical journals) were quantified by calculating the increase factor based on trials per year or the absolute differences and the associated 95% confidence intervals (CI), respectively.

The interobserver agreement between the reviewers of the reports was estimated for several essential criteria by Fleiss’ Kappa [17]. The agreement was between 0.27 and 1.0 (statement of non-inferiority or equivalence margin 1.0 (calculation of CI according to Fleiss not possible), justification of margin 0.54 (95% CI 0.17 to 0.59), sample size calculation based on a margin of 0.61 (95% CI 0.32 to 0.9), report of both per-protocol (PP) and intention-to-treat (ITT) analysis 0.27 (95% CI 0.16 to 0.38), presentation of the CI for the difference between treatment groups of 0.61 (0.32 to 0.9).

All analyses were carried out with the software SAS version 9.2 (SAS Institute Inc., Cary, NC, USA).

## Results

### Identification of reports

The literature search provided a total of 869 citations. A subset of 294 potentially relevant articles was identified by screening the titles and abstracts. After reviewing the full text articles we identified 209 primary reports; of these 167 (80%) were non-inferiority trials, and 42 (20%) were equivalence trials. The flowchart (Figure 1) gives an overview of the selection process. The most frequent causes for exclusion from the analysis were that the trial was not a non-inferiority or equivalence trial and was not a randomised clinical trial, which was mostly due to mentioning the terms ‘equivalence’ and ‘random’ in a different context, or for descriptive purpose only.

### Characteristics of reports

Table 2 provides information on characteristics of the non-inferiority and equivalence trials included in the analysis. Eighty reports (38%) were published in general medical journals and 129 reports (62%) in speciality journals. More than three-quarters of the trials compared two treatment groups. Thirty-one trials (15%) investigated three groups and only 15 (7%) investigated four groups. Twenty-five trials (12%) included a placebo arm to show non-inferiority or equivalence of one or two experimental treatments versus placebo (ten), to show superiority of one or two treatments compared by non-inferiority or equivalence analysis (eleven) or to investigate another objective (four).

**Table 2:** **Characteristics of reports of randomised non**-**inferiority and equivalent trials published in 2009**

		**Trial type**	
	**All trials** (**n** = **209**)	**Non**-**inferiority** (**n** = **167**)	**Equivalence** (**n** = **42**)
**Journal type**:			
General medical journal	80 (38)	68 (41)	12 (29)
Speciality journal	129 (62)	99 (59)	30 (71)
**Number of treatment groups**			
Two	163 (78)	130 (78)	33 (79)
Three	31 (15)	26 (16)	5 (12)
Four	15 (7)	11 (7)	4 (10)
**Placebo arm**			
Inclusion of a placebo arm	25 (12)	22 (13)	3 (7)
To show non-inferiority or equivalence of	10 (5)	8 (5)	2 (5)
1 or 2 experimental treatments v placebo			
To show superiority of 1 or 2 treatments	11 (5)	10 (6)	1 (2)
compared by non-inferiority or equivalence			
analysis			
**Sample size**			
Median per trial (IQR)	338 (174, 686)	379 (202, 714)	198 (90, 424)
**Comparison**			
Different treatments*	142 (68)	121 (73)	21 (50)
Same treatments*	80 (38)	59 (35)	21 (50)
Two strategies	49 (23)	32 (19)	17 (41)
Two doses	27 (13)	24 (14)	3 (7)
Two durations	5 (2)	3 (2)	2 (5)

Results are presented as number (%) unless stated otherwise. *Multiple answers possible, IQR: interquartile range.

Two-thirds of the reports (n = 142, 68%) dealt with trials that investigated different modalities of treatments. Eighty reports were of trials comparing the same pharmacological treatments but using different strategies (n = 49, 23%), doses (n = 27, 13%) or treatment durations (n = 5, 2%). Due to trials with more than two groups, some reports referred to the investigation of different as well as the same treatment types (n = 13, 6%). Five reports described trials with a cross-over design (2%).

In 104 of the trials reported, a binary endpoint was chosen as the primary endpoint (50%). A continuous endpoint was investigated in 90 reports (43%) and a time-to-event endpoint in 15 reports (7%).

The median number of patients randomised per trial was 338 (25^th ^to 75^th ^percentiles 174 to 686). Non-inferiority trials had a higher sample size than equivalence trials with 379 (202 to 714) v 198 (90 to 424).

### Reporting quality

Table 3 shows the percentage of non-inferiority and equivalence trials that met the criteria recommended in the CONSORT extension related to reporting and methodological quality as well as the assessment of the conclusion given by the authors. Just over half of the articles gave information on the method of randomisation (n = 115, 55%), whereas 190 articles reported on the method of blinding (91%). Almost half the reports were stated as double blind (43%), and more than one-third were described as open label (37%). Dates defining the period of recruitment were frequently presented (62%), but dates defining the period of follow up were only given in 10% of the reports. The flow of participants through the trial was presented in 146 (70%) of the reports with more non-inferiority trials than equivalence trials (73% v 57%) following this recommendation.

**Table 3:** **Compliance with criteria for reporting and methodology for non**-**inferiority and equivalence trials presented as number (%)**

	**All trials** (**n** = **209**)	**Non**-**inferiority** (**n** = **167**)	**Equivalence** (**n** = **42**)
**Criteria related to reporting quality generally important for randomised trials**			
Method of randomisation reported	115 (55)	94 (56)	21 (50)
Restriction method reported			
Blocking	59 (28)	45 (27)	14 (33)
Stratification	88 (42)	73 (44)	15 (36)
Minimisation	11 (5)	10 (6)	1 (2)
Method of blinding reported	190 (91)	152 (91)	38 (91)
Single blind	23 (11)	18 (11)	5 (12)
Double blind	90 (43)	73 (44)	17 (41)
Not blinded	77 (37)	61 (37)	16 (38)
Double dummy design	39 (19)	35 (21)	4 (10)
Blinding of administrators reported	30 (14)	26 (16)	4 (10)
Blinding of outcome assessor reported	57 (27)	46 (28)	11 (26)
Dates defining period of patient recruitment	130 (62)	104 (62)	26 (62)
Dates defining period of follow-up reported	20 (10)	16 (10)	4 (10)
Flow of participants presented as diagram	146 (70)	122 (73)	24 (57)
Baseline information presented for each group	201 (96)	162 (97)	39 (93)
Adverse events reported	159 (76)	133 (80)	26 (62)
**Criteria related to reporting quality particularly important for non**-**inferiority and equivalence trials**			
Clearly identified as non-inferiority or equivalence trial in title or abstract	175 (84)	139 (83)	36 (86)
Justification for using non-inferiority or equivalence design reported	101 (48)	80 (48)	21 (50)
Hypothesis stated clearly (text or formula)	104 (50)	85 (51)	19 (45)
Primary outcome identified clearly	196 (94)	159 (95)	37 (88)
Sample size calculation reported	187 (90)	151 (90)	36 (86)
All elements for recalculation of sample size reported	124 (59)	104 (62)	20 (48)
Justification of margin reported	51 (24)	38 (23)	13 (31)
Justification of margin reported by			
statistical considerations only	5 (2)	4 (2)	1 (2)
Justification of margin reported by clinical considerations only	31 (15)	23 (14)	8 (19)
Justification of margin reported by statistical as well as clinical considerations or results of a previous study	15 (7)	11 (7)	4 (10)
Statistical methods used for comparison reported	184 (88)	149 (89)	35 (83)
Analysis sets reported	188 (90)	156 (93)	32 (76)
**Criteria related to methodological quality of non**-**inferiority and equivalence trials**			
Non-inferiority or equivalence margin defined	197 (94)	161 (96)	36 (86)
Sample size taking into account the margin	163 (78)	136 (81)	27 (64)
Results reported using confidence interval	177 (85)	142 (85)	35 (83)
Figure showing confidence intervals and margins	34 (16)	24 (14)	10 (24)
Both per-protocol and ITT/modified ITT reported	87 (42)	74 (44)	13 (31)
**Interpretation of results given in the reports**			
Interpretation referring to results presented			
Comprehensible and accurate	165 (79)	135 (81)	30 (71)
Wrong	14 (7)	11 (7)	3 (7)
Incomprehensible	30 (14)	21 (13)	9 (21)
Statement on expected advantage	70 (34)	59 (35)	11 (26)
Expected advantage confirmed by results	52 (25)	46 (28)	6 (14)

ITT, intention-to-treat.

The majority of reports provided baseline information for each group (96%). In reports on equivalence trials the percentage was only slightly lower than in reports on non-inferiority trials (93% v 97%). Adverse events were presented in three-quarters of reports with a far higher percentage for non-inferiority trials (80%) than for equivalence trials (62%).

The criteria that are particularly important for non-inferiority and equivalent trials in relation to reporting quality were only followed in part. Most of the reported trials could be identified as non-inferiority or equivalence trial based on title or abstract (84%), but justification for the design (48%) or a clear hypothesis (50%) was only stated in half of the reports. Justification for the choice of the non-inferiority or equivalent margin was given in only 24% of reports. The majority of reports (94%) identified a clear primary endpoint, gave information regarding the sample size calculation (90%) and the statistical methods used for the group comparison (94%) as well as the analysis sets (90%). Mostly, the percentage of non-inferiority trials meeting the respective criteria was higher than the percentage of equivalence trials. An exception was the justification for the margin which was stated more often in reports for equivalence trials than for non-inferiority trials (31% v 23%).

### Methodological quality

The evaluation of the criteria related to methodological quality showed a high percentage of reports defining the non-inferiority or equivalence margin (96% v 86%). The defined margin was taken into account in the sample size calculation in 81% of the non-inferiority trials and 64% of the equivalence trials. Eighty-five reports presented results using a CI, but only 16% gave a graphical display of the CI together with the margin as recommended. Less than half of the reports (42%) stated the results of the ITT as well as the PP analysis; more non-inferiority trials than equivalence met this criterion (44% v 31%).

All reports presented an interpretation of the trial results. The conclusion drawn by the authors was comprehensible and accurate in 165 reports (79%). The assessment showed a higher percentage of non-inferiority trials than equivalence trials giving a correct interpretation (81% v 71%). In total, the interpretation was wrong in 14 reports (7%), and in 30 reports (14%) it was incomprehensible in the light of the results presented. The percentage of equivalence trials presenting an incomprehensible conclusion was considerably higher than in non-inferiority trials (43% v 13%). A third of the reports (34%) gave a more detailed statement, on which advantage was to be expected as compensation for a potential although irrelevant inferiority. In 52 reports (25%) this statement could be confirmed by the trial results. However, the reported advantage could frequently not be verified by the reviewers due to lack of information given in the report (for example, actual reduction of costs).

Relevant guidelines were quoted only rarely. The CONSORT extension was quoted in 16 reports (8%). The CONSORT Statement for randomised trials was quoted only twice, and the Points-to-Consider-document referring to the choice of the margin was referenced only four times.

### Changes in trial characteristics and quality of reporting

**Figure 2:**
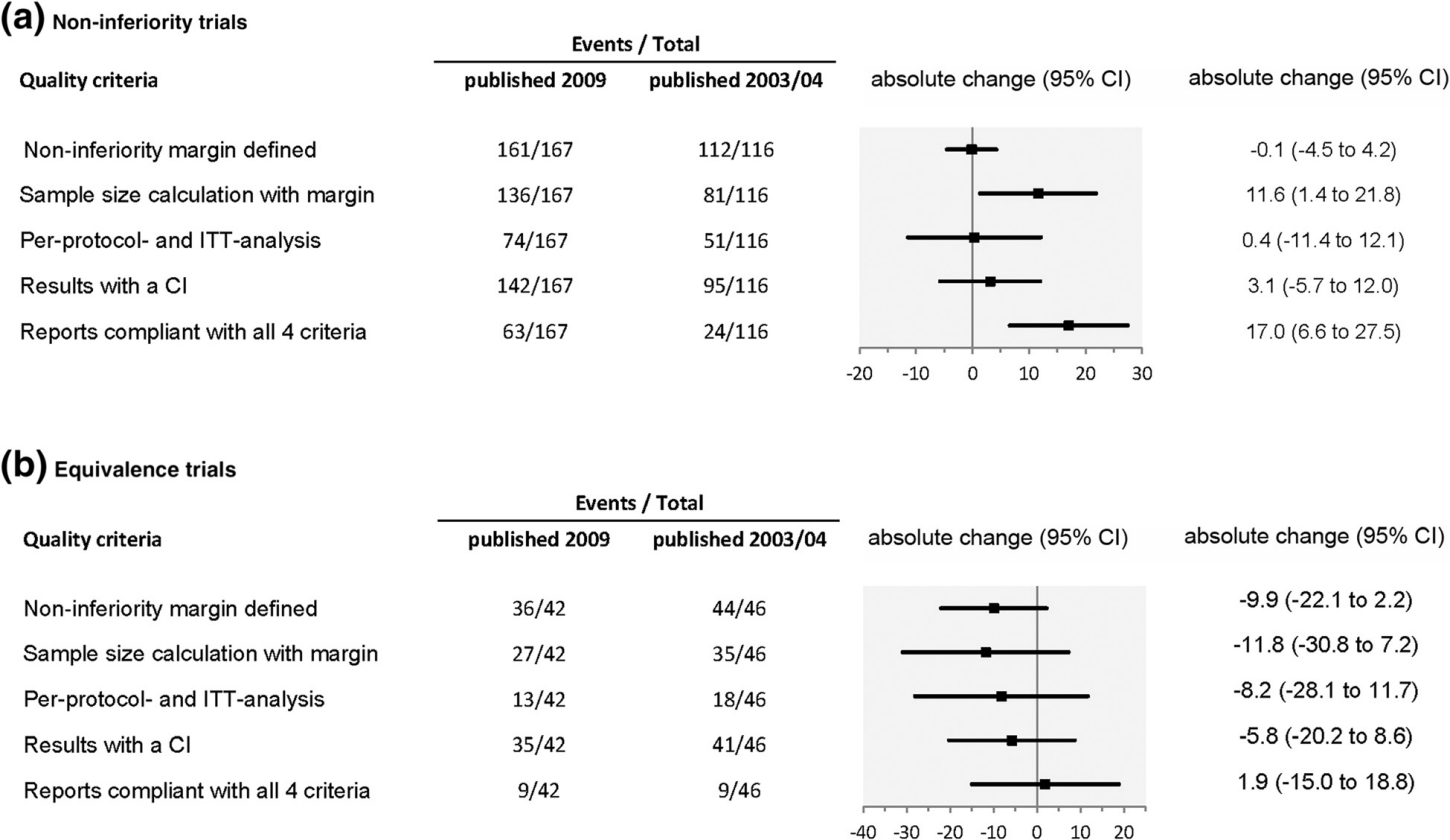
**Change of adherence to quality criteria for reporting of non**-**inferiority (a) and equivalence (b) trials published after release of the CONSORT extension for non-inferiority and equivalence trials in relation to trials published before the CONSORT extension **[10].

The comparison of our findings with the results of a previous study published by Le Henanff *et al*. [10] before the CONSORT extension was released is shown in Table 4 and Figure 2. The previous study included 162 articles that were identified within two years (116 non-inferiority reports, 46 equivalence reports; Table 4, Figure 2, [10]), whereas in 2009, a total of 209 articles was identified (167 non-inferiority reports, 42 equivalence reports). This corresponds to an increase by a factor of 2.9 for non-inferiority trials and by 1.8 for equivalence trials published per year. Furthermore, the number of non-inferiority or equivalence trials that included a placebo group increased from 11 trials in 2003 and 2004 to 25 trials in 2009 (increase by a factor of 4.5). With regard to the type of comparison, a larger proportion of trials in 2009 compared the same treatments (increase by a factor of 7).

**Table 4:** **Changes of trial characteristics**^**a **^**and adherence**^**b **^**to criteria related to reporting and methological quality of non**-**inferiority and equivalence trials reported before and after publication of the CONSORT extension**

	**Increase factor based on trials per year** (**2009 to 2003**/**4**)		
**Trial characteristics**	**All trials**	**Non**-**inferiority**	**Equivalence**
Number of trials reported per year	2.6 (2.1, 3.2)	2.9 (2.3, 3.7)	1.8 (1.2, 2.8)
Inclusion of a placebo arm	4.6 (2.2, 10.2)	5.5 (2.4, 14.3)	2.0 (0.3, 14.9)
Comparison of different treatments	2.0 (1.6, 2.6)	2.4 (1.8, 3.1)	1.1 (0.6, 2.0)
Comparison of same treatments	7.0 (4.3, 11.6)	8.4 (4.7, 16.4)	4.7 (2.1, 11.6)
Two strategies	6.1 (3.4, 11.5)	7.1 (3.3, 16.9)	4.9 1.9, 13.9)
Two doses	18.0 (5.5, 92.7)	24.0 (6.0, 209.5)	6.0 (0.5, 315.0)
Two durations	2.5 (0.5, 12.6)	1.3 (0.1, 11.6)	4.0 (0.2, 236.0)
	**Absolute change in % (95% CI) of adherence (2009 to 2003/4)**		
**Criteria related to reporting quality**	**All trials**	**Non**-**inferiority**	**Equivalence**
Clearly identified as non-inferiority or equivalence trial in title or abstract	−15.6 (−20.8, -10.5)	na	na
Trial described as double blind	−15.0 (20.8, 10.5)	−12.3 (−24.1, -0.6)	−13.0 (−33.6, 7.5)
Justification of margin reported	4.0 (−4.5, 12.5)	2.1 (−7.7, 11.8)	11.4 (−6.7, 29.5)
Sample size calculation reported	11.1 (3.5, 18.7)	11.1 (2.5, 19.7)	9.6 (−6.6, 25.9)
All elements for recalculation of sample size reported	5.6 (−4.5, 15.8)	8.0 (−3.7, 19.6)	−4.6 (−25.5, 16.3)
**Criteria related to methodological quality**			
Non-inferiority or equivalence margin defined	−2.0 (−6.3, 2.3)	−0.1 (−4.5, 4.2)	−9.9 (−22.1, 2.2)
Sample size calculation taking into account the margin	6.4 (−2.6, 15.3)	11.6 (1.4, 21.8)	−11.8 (−30.8, 7.2)
Results reported using confidence interval	0.7 (−6.7, 6.2)	3.1 (−5.7, 12.0)	−5.8 (−20.2, 8.6)
Both per-protocol and ITT/modified ITT-analysis reported	−1.0 (−11.1, 9.2)	0.4 (−11.4, 12.1)	−8.2 (−28.1, 11.7)
All 4 methodological quality criteria fulfilled	14.1 (5.1, 23.0)	17.0 (6.6, 27.5)	1.9 (−15.0, 18.8)

^a^Increase factor based on trials per year (95% CI assuming Poisson counts); ^b^absolute change in % (95% CI). ITT, intention-to-treat; na, not applicable as no subgroup results were reported for 2003/04.

The comparison of the adherence to criteria related to reporting quality showed changes varying considerably across the different topics. The proportion of non-inferiority and equivalence trials, which were clearly identifiable in the title or abstract, decreased by 15% from almost 100% down to 84%. The proportion of trials described as double blind also decreased by 15% (from 58% to 43%).

The percentage of reports presenting a justification of the defined margin continued to remain low (change from 20% to 24%), whereas the proportion of reports that presented a sample size calculation increased by 11%. Reports on non-inferiority trials more often showed all elements for recalculation of the sample size (increase by 8%), but this proportion decreased for equivalence trials (5%).

Considering the methodological quality there was hardly any improvement. Reports of non-inferiority trials published after release of the CONSORT extension more often presented a sample size calculation taking into account the margin, as compared to reports previously published, with an absolute change of 12% (1.4% to 21.8%). The adherence to other methodological items, such as the defined margin presented, results of PP and ITT analysis presented, and results presented with a CI, showed almost no changes (absolute change in percentage of adherence was between −0.1% and 3.1%). Only the percentage of reports on non-inferiority trials compliant with all four important methodological criteria increased by 17 percentage points (6.6 to 27.5).

Reports on equivalence trials showed a more negative picture. The adherence to the methodological criteria decreased as compared to reports published before the CONSORT extension. The absolute change varied between −12% and −6%. Only the proportion of reports that were compliant with all important methodological criteria remained stable at a level of 20%, with absolute change of 1.9% (−15% to 18.8%).

## Discussion

We investigated standard criteria regarding the reporting and methodological quality of non-inferiority and equivalence trials recommended in the CONSORT extension and compared our results with the results of a previous investigation including trials reported before the CONSORT extension was published [10,12].

The major result of our investigation was a substantial lack of quality in reporting and conduct. Most of the reported trials could be identified as non-inferiority or equivalence trials based on title or abstract, but less than half of the reports gave a rationale for the design or the expected advantage. The non-inferiority or equivalence margin was defined in most reports, but the majority of reports gave no information about the justification of the margin defined. A sample size calculation was presented in 90% of the reports, but it did not always take the margin into account. More than half of the reports did not present the results of ITT as well as PP analyses. Far too many reports gave an interpretation that was wrong or incomprehensible. Reports of equivalence trials mostly showed a lower reporting quality than reports of non-inferiority trials. Overall, there is no relevant improvement since the release of the CONSORT extension for non-inferiority and equivalence trials. Moreover, the need to improve certain aspects also comprised items that should be standard quality for any randomised trial.

However, this overall finding might be biased due to an increased number of trials published in journals that do not have a rigorous peer-review process based on strict rules, and that do not endorse the CONSORT statement and its extensions.

Hopewell and colleagues showed that journal endorsement of CONSORT seems to be associated with improvements in reporting of randomised trials [18]. They highlighted the need for journals to endorse the CONSORT statement and its extensions for special designs and to incorporate the checklists and recommendations into the peer-reviewing process [18]. In 2006, Hopewell *et al*. [8] found a proportion of 44% randomised trials published in journals that endorsed CONSORT. Later in 2008, Hopewell and colleagues [18] reported that an even smaller proportion of a selection of high-impact journals mentioned the general CONSORT statement in the instructions for authors, and that only a fraction of those journals stated that this was a requirement. Moreover, very few journals mentioned the CONSORT extension papers [18]. Since neither the present nor the previous study determined the proportion of reports published in CONSORT-endorsing journals, we could not give information referring to this proportion in journals publishing non-inferiority and equivalence trials. Instead, we stratified by impact factor and journal type and compared the results for reports published in high-impact general medical journals, in low-factor general medical journals, and in speciality journals (see Additional file 1, Annex). The number of non-inferiority and equivalence trials that have been published per year increased strongly. Here, it should be noted that reports published in speciality medical journals are still predominating in our investigation, but the proportion is somewhat smaller than in the previous study what might be caused by a different classification.

In our review we found that only a little over half the reports gave information referring to the randomisation. The proportion was substantially larger in high-impact journals as compared to low-impact general medical journals or speciality journals. Since a similar composition for the groups compared is best attained by randomly dividing a single sample population, and randomisation is a sound basic for statistical inference [4], it should be implemented and the method should be reported.

We found that more than half of the articles reported blinding of the trial and more than one-third were open label trials. Hopewell et al. [8] found a similar proportion of randomised trials referring to any blinding but a smaller proportion were reported as unblinded. However, they also found a substantial proportion of reports that do not give clear information on blinding. In the evaluation of non-inferiority trials by Wangge *et al*. [14] there one third of trials were also reported as open label. In their review they pointed out that this was not consistent with the guidelines, which recommend blinding of any randomised trial whenever possible [4].

A diagram showing the flow of participants for each group was presented in more than two-thirds of the reports on non-inferiority and equivalence trials. The subgroup of high-impact journals showed even a larger proportion. A review on randomised trials in special medical fields found smaller proportions [19,20]. Surprisingly, Hopewell *et al*. [8] reported that fewer than one third of reports of randomised trials published in 2006 included details of participant flow. Overall, with respect to those items there seemed to be a somewhat better reporting quality in non-inferiority and equivalence trials compared to any randomised trials.

The most important specific element in planning a non-inferiority or equivalence trial is the definition and the method of the determination of the margin. In non-inferiority trials the margin was reported in most of the articles, which was much more often than in equivalence trials. In comparison to the results of Le Henanff *et al*. this was no improvement, and was even a change for the worse in equivalence trials. Only reports published in high-impact general medical journals accomplished this requirement in full. However, the far more important justification of the margin was just given in a quarter of the reports. Though this meant a small improvement compared to the period before the release of the CONSORT extension [12], it was far too low. Wangge *et al*. reported a higher percentage of 46% of the articles that gave the method by which the margin was determined, but this was also not sufficient [14]. This higher percentage could be caused by the different selection of trials the authors investigated, which excluded non-drug trials. However, we did not find such results for trials published in high-impact general medical journals.

Most of the reports presented information on the sample size calculation and there was some improvement in comparison to previous studies. Although this portion was higher in high-impact than in low-impact journals, the more important finding was the fact that in a substantial proportion of reports the margin was not considered, and an even larger number of articles did not report on all elements needed for a recalculation (20% or 40%, respectively). There too, in high-impact journals we found a better reporting quality with respect to the details than in speciality or low-impact journals. The lower quality in speciality journals was also confirmed by a review on non-inferiority and equivalence trials in a specialised medical area [13].

With respect to the analysis, the guidelines state that both ITT and PP analysis have equal importance in non-inferiority and equivalence trials [5,12], since both analyses can be biased. However, nearly half of the reports stated the results of both analyses, which is similar to the percentage in the period before publication of the CONSORT extension [10]. Trials published in high-impact journals showed only slightly better results. However, the overall results found by Wangge *et al*. were similar, though the proportion of high-impact journals reporting on both analysis sets was considerably lower [14].

In most reports the results were presented with a CI as recommended (85%). In high-impact journals this was even true for 100% of the reports. Nevertheless, only a fraction of reports graphically displayed the CI together with the margin, which is the recommended and most informative way of presenting the results with respect to interpretation. In our investigation there were only small differences between trials published in the different types of journals. But in the speciality reports reviewed by Eyawo *et al*., graphical display of results was very rare [13].

In nearly two-thirds of the reports the authors concluded that non-inferiority or equivalence, respectively, was demonstrated. Wangge *et al*. found a substantially higher portion of the reports claiming to have demonstrated non-inferiority (90%) [14]. Nevertheless, more important than the respective conclusion by the authors is whether the conclusion is confirmed by the results presented. The percentage of reports with wrong or incomprehensible conclusion added up to one fifth for non-inferiority trials or even more for equivalence trials, which is far too high. After all, many readers gather only the primary message of a paper and will be misled.

The strengths of our investigation were that we assessed the complete set of reports of non-inferiority and equivalence trials published in 2009 and identified these by a clearly defined search strategy. Hence, the basis of our investigation is the entire picture of published reports and considerably exceeds a more or less representative random sample [11,14] or an analysis of trials regarding only a specific therapeutic area [13,19-22].

In order to check the criteria most relevant for non-inferiority and equivalence trials, we abstracted most of the recommendations described in the extension to the CONSORT statement for this trial type. Furthermore, we defined all evaluation criteria a priori and established a comprehensive review procedure.

However, our research has also several limitations. Due to the considerable effort we could only investigate reports published in 2009 and we therefore had no own investigation of another year’s set of reports for a direct comparison. We decided to use a search strategy similar to the one used in a previous survey published in 2006 [10] to allow a comparison with this and to investigate the impact of the CONSORT extension. However, we are aware of the possible bias due to the course of time and different reviewers. This approach was limited due to the selection of criteria reported in the previous publication [10] and did not allow the comparison of some important items as well as a stratified comparison for high- and low-impact general medical journals for both years. We therefore could only present the stratified results for reports published in 2009.

Moreover, it was not possible to standardise or abstract all 22 items referred to in the CONSORT statement. For example, the appropriateness of the interventions with respect to trials that established efficacy for the reference treatment is an important issue, but this would have required the assessment of a huge number of associated trials published elsewhere. Due to the inclusion in our database search of the term ‘equivalent’ we got a particularly high number of unspecific results. Though each abstract was carefully examined and questionable cases were clarified by two reviewers using the full text, it might be possible that some further suitable reports were not selected for the analysis.

## Conclusion

There are still important deficiencies in the reporting on the methodological approach as well as on results and interpretation of non-inferiority and equivalence trials even in high-impact journals. Improvement of the overall situation seems to require other measures than appropriate guidelines and recommendations. It might be helpful to facilitate a better overview and access to the guidelines relevant for the different trial types. But it might be more important to support researchers and reviewers by offering specific training accompanied by an explicit demand of a strict monitoring of CONSORT requirements in the peer-review process. This approach is strongly assisted by the EQUATOR-network, an international initiative that tries to improve the reliability and value of medical research literature by promoting transparent and accurate reporting of research studies [23,24]. Hopefully, these comprehensive measures will have a positive effect on the quality of reporting in different trial types. In any case, there is an urgent need for improvement, which is especially important against the background of the strongly increased number and relevance of non-inferiority and equivalence trials during the past years.

## Competing interests

All authors declare that they have no competing interests.

## Authors’ contributions

All authors contributed to the concept and design of the study. PS, NB, MN collected the data and conducted the analysis. All authors contributed to the interpretation of the data. PS drafted the article. All authors commented on the first draft, revised the manuscript critically for important intellectual content, and approved the final version.

## Supplementary Material

Additional file 1 Annex. Click here for file
