# Supplementary material for: Quality of reporting of clinical non-inferiority and equivalence randomised trials - update and extension
Source: Trials. 2012 Nov 16;13:214. doi: 10.1186/1745-6215-13-214 (PMC3554513; doi:10.1186/1745-6215-13-214)
Supplement: Additional file 1 — Annex. [file 1745-6215-13-214-S1.doc]

Annex

Figures

Figure A1: Change of adherence to quality criteria for reporting of non-inferiority and equivalence trials published in high-impact and low-impact general medical journals after release of the CONSORT extension for non-inferiority and equivalence trials in relation to trials published before [10]


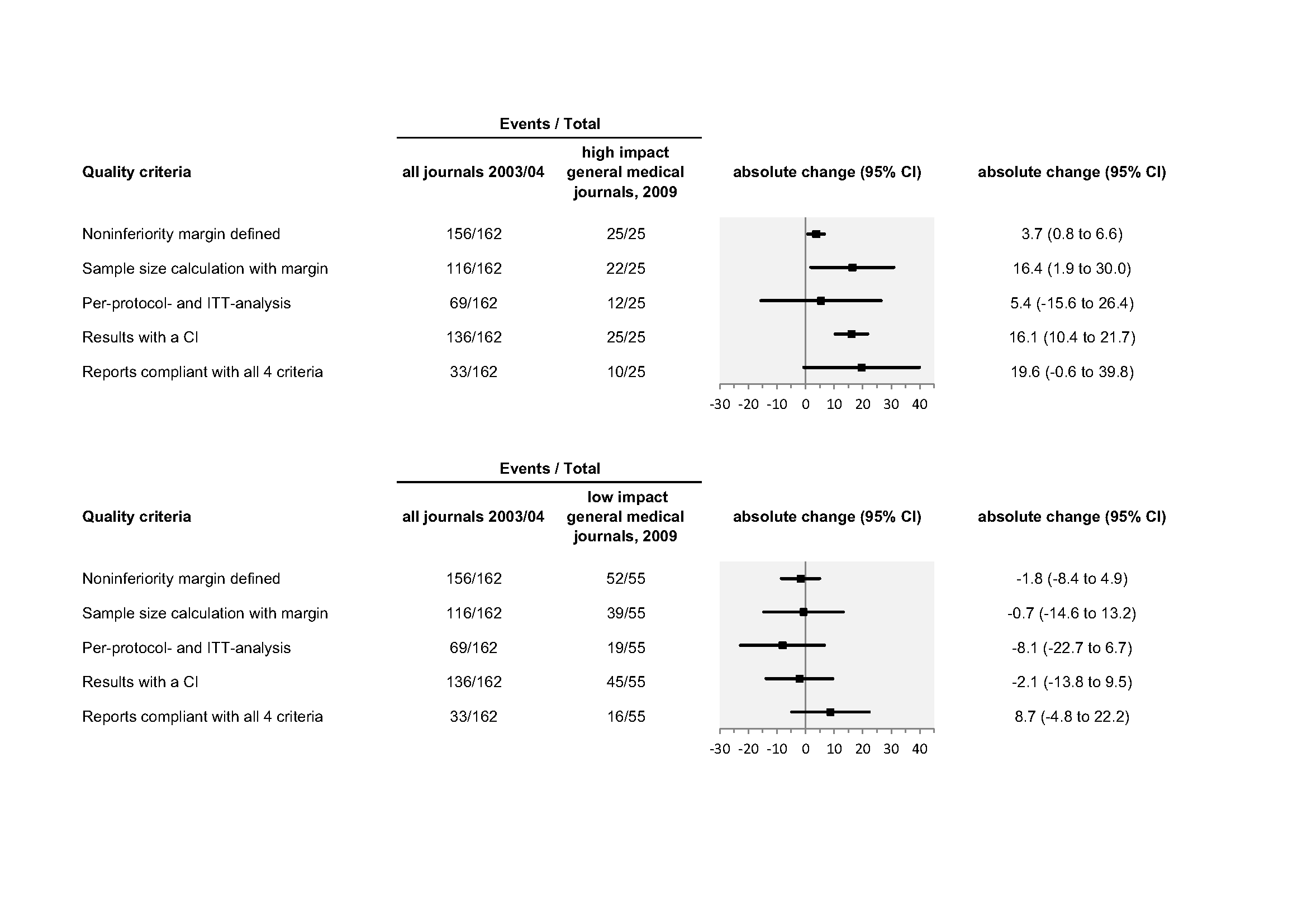


Tables

Table A1: Compliance with criteria for reporting and methodology for non-inferiority and equivalence trials published in general medical journals separated by impact factor and specialty journals (number (%))

|  |  | **High impact factor general journals  (n = 25)** | **Low impact factor general journals  (n = 55)** | **Specialty journals (n=129)** |
| --- | --- | --- | --- | --- |
| **Trial design** | | | | |
| Non-inferiority trials |  | 21 (84) | 47 (85) | 99 (77) |
| Equivalence |  | 4 (16) | 8 (15) | 30 (23) |
| **Criteria related to reporting quality generally important for randomised trials** | | | | |
| Method of randomisation reported |  | 21 (84) | 33 (60) | 61 (53) |
| Restriction method reported |  |  |  |  |
| Blocking |  | 8 (32) | 22 (40) | 29 (23) |
| Stratification |  | 17 (68) | 23 (42) | 48 (37) |
| Minimisation |  | 7 (28) | 3 (5) | 1 (1) |
| Method of blinding reported as |  | 22 (88) | 52 (95) | 116 (90) |
| Single blind |  | 2 (8) | 4 (7) | 17 (13) |
| Double blind |  | 9 (36) | 24 (44) | 57 (44) |
| Not blinded |  | 11 (44) | 24 (44) | 42 (33) |
| Double dummy design |  | 6 (24) | 10 (18) | 23 (18) |
| Blinding of administrators reported |  | 0 (0) | 14 (25) | 15 (20) |
| Blinding of outcome assessor reported |  | 5 (20) | 22 (40) | 25 (34) |
| Dates defining period of patient recruitment |  | 20 (80) | 36 (65) | 74 (57) |
| Dates defining period of follow-up reported |  | 8 (32) | 3 (5) | 9 (7) |
| Flow of participants presented as diagram |  | 21 (84) | 41 (75) | 84 (65) |
| Baseline information presented for each group |  | 24 (96) | 52 (95) | 125 (97) |
| Adverse events reported |  | 20 (80) | 37 (67) | 102 (79) |
| **Criteria related to reporting quality particularly important for non-inferiority and equivalence trials** | | | | |
| Clearly identified as non-inferiority or equivalence trial in title or abstract |  | 24 (96) | 39 (71) | 112 (87) |
| Justification for using non-inferiority or equivalence design reported |  | 9 (36) | 30 (64) | 58 (45) |
| Hypothesis stated clearly (text or formula) |  | 17 (68) | 19 (35) | 68 (53) |
| Primary outcome identified clearly |  | 24 (96) | 52 (95) | 120 (93) |
| Sample size calculation reported |  | 25 (100) | 48 (87) | 114 (88) |
| All elements for recalculation of sample size reported |  | 15 (60) | 33 (60) | 84 (65) |
| Justification of margin reported |  | 6 (24) | 18 (33) | 27 (21) |
| Justification of margin by |  |  |  |  |
| statistical considerations only |  | 0 (0) | 2 (4) | 3 (2) |
| clinical considerations only |  | 1 (4) | 11 (20) | 19 (15) |
| statistical as well as clinical considerations or   results of a previous study |  | 5 (20) | 5 (9) | 5 (12) |
| Statistical methods used for comparison reported |  | 25 (100) | 47 (85) | 121 (94) |
| Analysis sets reported |  | 18 (72) | 39 (71) | 86 (67) |
| **Criteria related to methodological quality of non-inferiority and equivalence trials** | | | | |
| Non-inferiority or equivalence margin defined |  | 25 (100) | 52 (95) | 120 (93) |
| Sample size taking into account the margin |  | 22 (88) | 39 (71) | 100 (78) |
| Results reported using confidence interval |  | 25 (100) | 45 (82) | 105 (81) |
| Figure showing confidence intervals and margins |  | 5 (20) | 11 (20) | 18 (17) |
| Both per-protocol and ITT/modified ITT reported |  | 12 (48) | 19 (35) | 55 (43) |
| **Interpretation of results given in the reports** | | | | |
| Interpretation referring to results presented |  |  |  |  |
| Comprehensible and accurate |  | 25 (100) | 39 (71) | 101 (78) |
| Wrong |  | 0 (0) | 6 (11) | 8 (6) |
| Incomprehensible |  | 0 (0) | 10 (18) | 20 (16) |
| Statement on expected advantage |  | 9 (36) | 16 (29) | 45 (35) |
| Expected advantage confirmed by results |  | 7 (28) | 14 (25) | 31 (24) |
